# Supplementary material for: Optical nanoscopy of transient states in condensed matter
Source: Sci Rep. 2015 Jul 28;5:12582. doi: 10.1038/srep12582 (PMC4648477; doi:10.1038/srep12582)
Supplement: Supplementary Information [file srep12582-s1.pdf]

Optical nanoscopy of transient states in condensed matter

F. Kuschewski [1], S.C. Kehr [1], B. Green [2],
Ch. Bauer [2,3], M. Gensch [2], and L.M. Eng [1]

[1] Institut für Angewandte Physik, TU Dresden, Dresden, Germany
[2] Helmholtz-Zentrum Dresden-Rossendorf, Germany
[3] Freie Universität Berlin, Berlin, Germany

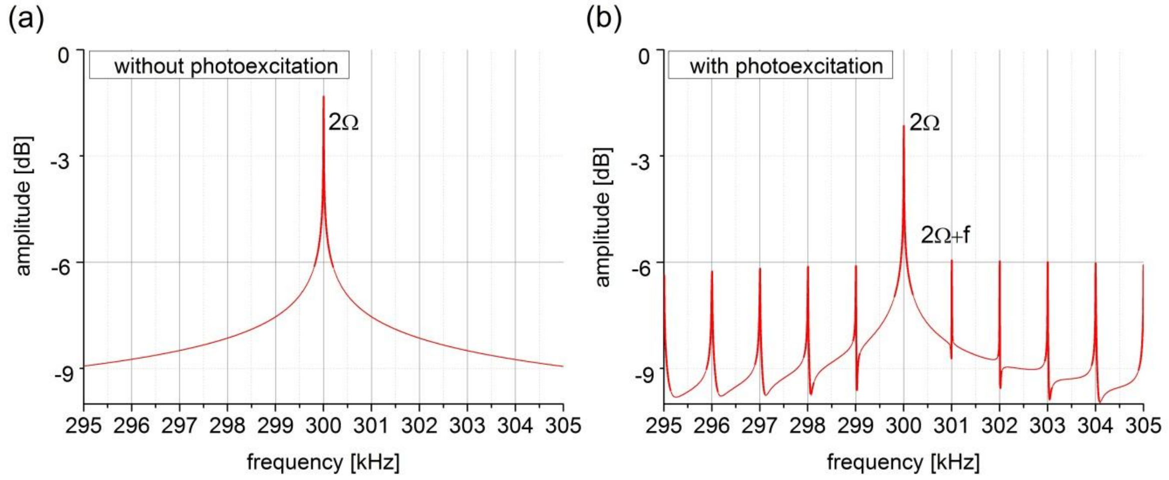

Supplementary Figure S1 | Numerical simulation of the sideband generation. The expected near-field signal of a germanium sample is calculated with the dipole-model[33], assuming a spherical metal tip (radius r = 10 nm) that oscillates close to the sample surface with an amplitude of A = 50 nm at a frequency of Ω = 150 kHz. The probe beam consists of a cw source with a wavelength of 10 µm. In order to describe the pumping effect, we assumed that the dielectric constant ε=16 of the germanium sample is modified via the electron-hole plasma by an additional Drude-like term. Here, the latter contribution has been described by charge carriers densities of electrons and holes of $n_e=n_h=3\times10^{19}$ $1/cm^3$ that decay exponentially with a decay time in the range of µs and that are periodically excited with a frequency of f=1 kHz. The Fourier-transformation of the resulting spatio-temporal near-field signal shows the spectra for the relaxated and transient state. (a) Without photo-excitation, Re(ε) = 16 is constant and the second harmonic signal $NF_{2\Omega}$ is observed at 2Ω = 300 kHz. (b) When pumping the sample with 1 kHz, Re(ε) is modulated and shows negative values for a short period of time. This leads to an amplitude modulation of the near-field signal and, consequently, to sidebands of the 2Ω peak at multiples of the pumping frequency f. Direct measurement of the first sideband yields a very high contrast between relaxed and photo excited state.

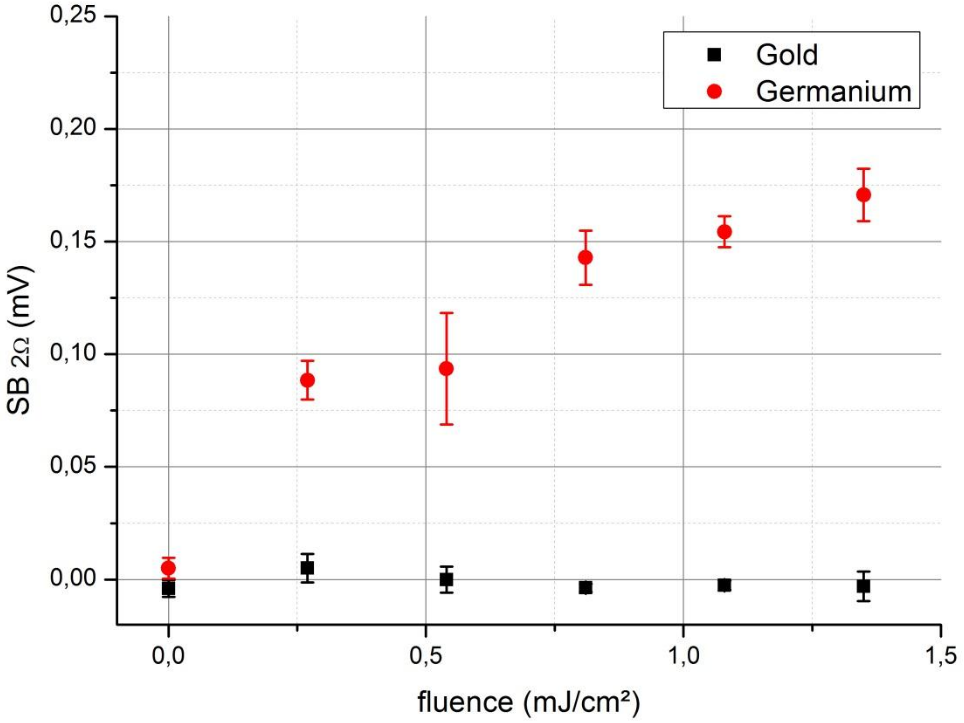

Supplementary Figure S2 | Excitation probed with FEL. Sideband signal measured on pure germanium and gold reference sample, being probed with the FEL (wavelength λ=10.6 µm, average power P_{Probe}=25 mW) and excited by the Nd:YAG pump laser (various fluence). The gold reference sample is not excited by the Nd:YAG laser, thus showing no pump-related effects, whereas the germanium sample shows increasing values in the sideband, consistent with previous measurements with the CO_2 laser (see main text). In contrast to the cw-operated CO_2 laser, the FEL is a pulsed infrared radiation source with a repetition rate of 13 MHz and a pulse length in the ps range. This measurement demonstrates that the novel demodulation technique is working properly also when probing with the pulsed FEL, enabling time-resolved measurements in the future.
